# Enhancement of Chaperone Activity of Plant-Specific Thioredoxin through γ-Ray Mediated Conformational Change

**DOI:** 10.3390/ijms161126019

**Published:** 2015-11-13

**Authors:** Seung Sik Lee, Hyun Suk Jung, Soo-Kwon Park, Eun Mi Lee, Sudhir Singh, Yuno Lee, Kyun Oh Lee, Sang Yeol Lee, Byung Yeoup Chung

**Affiliations:** 1 Research Division for Biotechnology, Advanced Radiation Technology Institute (ARTI), Korea Atomic Energy Research Institute (KAERI), 29 Geumgu-gil, Jeongeup 580-185, Korea; sslee@kaeri.re.kr (S.S.L.); neeunmi@nate.com (E.M.L.); sudhir4u@gmail.com (S.S.); 2 Department of Biochemistry, College of Natural Sciences, Kangwon National University, Chuncheon 200-701, Korea; hsjung@kangwon.ac.kr; 3 Crop Foundation Division, National Institute of Crop Science, Rural Development Administration, 181 Hyeoksin-ro, Iseo-myeon, Wanju-gun 565-851, Korea; sookwonpark@korea.kr; 4 Division of Applied Life Science (Brain Korea 21 Program), Gyeongsang National University, 501 Jinju-daero, Jinju 660-701, Korea; yunolee1@gmail.com (Y.L.); leeko@gnu.ac.kr (K.O.L.); sylee@gnu.ac.kr (S.Y.L.)

**Keywords:** chaperone, γ-ray, protein, structural change, thioredoxin

## Abstract

AtTDX, a thioredoxin-like plant-specific protein present in *Arabidospis* is a thermo-stable and multi-functional enzyme. This enzyme is known to act as a thioredoxin and as a molecular chaperone depending upon its oligomeric status. The present study examines the effects of γ-irradiation on the structural and functional changes of AtTDX. Holdase chaperone activity of AtTDX was increased and reached a maximum at 10 kGy of γ-irradiation and declined subsequently in a dose-dependent manner, together with no effect on foldase chaperone activity. However, thioredoxin activity decreased gradually with increasing irradiation. Electrophoresis and size exclusion chromatography analysis showed that AtTDX had a tendency to form high molecular weight (HMW) complexes after γ-irradiation and γ-ray-induced HMW complexes were tightly associated with a holdase chaperone activity. The hydrophobicity of AtTDX increased with an increase in irradiation dose till 20 kGy and thereafter decreased further. Analysis of the secondary structures of AtTDX using far UV-circular dichroism spectra revealed that the irradiation remarkably increased the exposure of β-sheets and random coils with a dramatic decrease in α-helices and turn elements in a dose-dependent manner. The data of the present study suggest that γ-irradiation may be a useful tool for increasing holdase chaperone activity without adversely affecting foldase chaperone activity of thioredoxin-like proteins.

## 1. Introduction

In oxygenated environments γ-irradiation can damage, inactivate, or change proteins either directly by breaking covalent bonds of proteins or indirectly by generating various reactive oxygen species (ROS). These toxic products such as hydrated electrons, hydrogen atoms, hydrogen peroxides, and hydroxyl radicals are primarily responsible for the protein damage [1,2,3,4,5]. Exposure of protein to γ rays induces conformational changes, oxidizes amino acids, breaks covalent bonds, and produces protein-free radicals [6,7,8,9,10]. Fragmentation, cross-linking, aggregation, and oxidation by oxygen radicals generated in radiolyzed water are some other chemical changes associated with irradiated proteins [6,7,8,9,10]. Thus, radiation generally leads to many irreversible changes at molecular level.

In our previous studies, radiation-mediated changes in the structure and function of the protein was recorded by determining the changes in the molecular properties of 2-cysteine peroxiredoxins (2-Cys Prxs), which have dual enzymatic functions of a peroxidase and a molecular chaperone [11,12,13,14]. Exposure of 2-Cys Prxs to ionizing radiations, such as, γ rays and electron beams, greatly increased the holdase chaperone activity increased by 3–4-fold compared to non-irradiated protein, but peroxidase activity declined with increasing radiation doses [12,13,14]. The changes in the functions were closely associated with its structural status, including high molecular weight (HMW) complex formation, fragmentation, increasing hydrophobicity, and secondary structural changes of the protein. These results led us to assume that ROS originating from γ-irradiation can rapidly attack active sites of enzymes or proteins. Active sites of enzymes, especially Prx, contain functional groups of amino acid residues such as a cysteine, which have the highest reactivity for redox reaction between a protein and its substrate and thus make it more vulnerable to the attack of ROS and in turn deactivation. Therefore, original enzymatic activity (peroxidase) of Prxs tend to decrease dose-dependently after γ-irradiation.

To determine whether this phenomenon, which was recently discovered by our group, is limited to 2-Cys Prxs, we examined a plant-specific thioredoxin-like protein, namely, *Arabidopsis thaliana* tetratricopeptide domain-containing thioredoxin (AtTDX). AtTDX belongs to the thioredoxin family II proteins fused with tetratricopeptide repeats (TPRs), and has been found only in *Arabidopsis*, rice, tobacco, and grape [15,16]. AtTDX encodes a two-domain 42 kDa protein consisting of 380 amino acids. The amino-terminal domain of AtTDX includes three TPR domains flanked by acidic and basic regions, whereas the carboxyl-terminal domain contains a thioredoxin domain [15,16]. AtTDX, located in the cytosol and nucleus of the cell is capable of performing multiple functions, such as, a disulfide reductase and as a molecular chaperone (both holdase and foldase) [16,17]. Multi-functionality of AtTDX mainly depends on its conformational status. Thioredoxin and foldase chaperone activities are mainly exhibited by low molecular weight forms (LMW; viz. monomeric, dimeric, tetrameric, and hexameric) of AtTDX, whereas holdase chaperone activity predominates in double-layered dodecamer, and globular-shaped HMW complexes [16]. These structural changes are triggered by heat stress leading to transformation from LMW to HMW complexes accompanied with change from thioredoxin and foldase chaperone to a holdase chaperone [16].

This study addresses the impact of γ-irradiation on enzymatic and structural properties of AtTDX and explains the enhanced holdase chaperone activity of modified AtTDX.

## 2. Results

### 2.1. Influence of γ Irradiation on Enzymatic Functions of AtTDX

Previously, we characterized the AtTDX from *Arabidopsis*, which showed multiple functions such as thioredoxin, holdase, and foldase chaperone [16]. These functions were regulated by the quaternary structure/oligomeric status of the protein. The HMW form shows high holdase chaperone activity, whereas the LMW form, mainly tetrameric, predominantly possesses strong thioredoxin and foldase chaperone activities.

**Figure 1:**
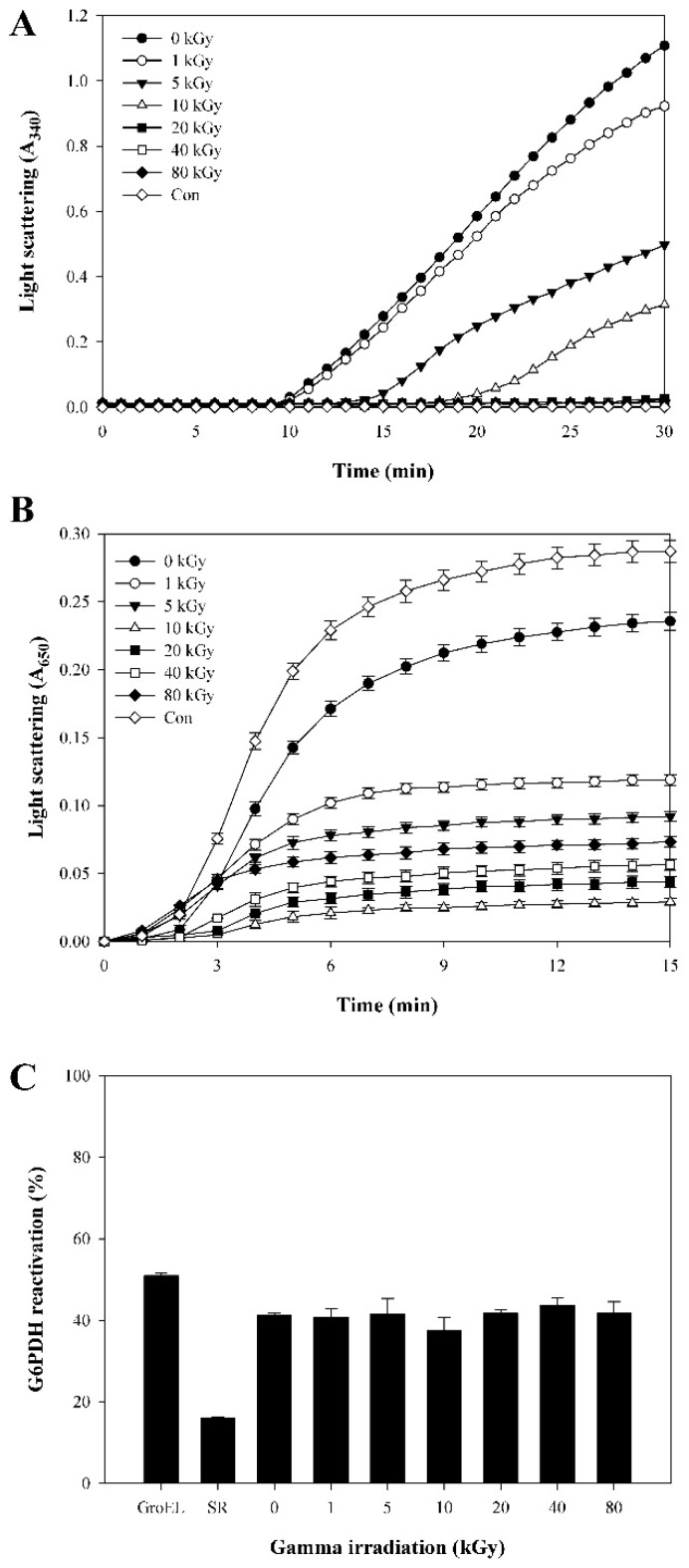
Comparison of thioredoxin, holdase, and foldase chaperone activities between irradiated and non-irradiated AtTDX. (**A**) Thioredoxin activities of irradiated and non-irradiated AtTDX (10 μM) were measured using insulin reduction assay. Control (Con) was measured in the absence of AtTDX protein; (**B**) Holdase chaperone activities of irradiated and non-irradiated AtTDX were measured using aggregation of MDH at 43 °C at a subunit molar ratio of 1 MDH/2 AtTDX. Con was measured in the absence of AtTDX protein. Data are the means of at least three independent experiments; (**C**) Foldase chaperone activity of irradiated and non-irradiated AtTDX. The Cys-free form of G6PDH was denatured in 4 M guanidine hydrochloride for 2.5 h and then allowed to refold in a renaturation buffer for 7 h in absence (spontaneous refolding; SR) or presence of 1 μM irradiated and non-irradiated AtTDX or 14 μM of the chaperone GroEL as a positive control. Data are the means of at least three independent experiments.

To evaluate the influence of γ-irradiation on AtTDX functions, protein was exposed to various doses of γ rays. Thioredoxin activity of irradiated AtTDX gradually decreased with the increasing doses leading to no activity at 20 kGy of γ-irradiation (Figure 1A). Increasing amounts of AtTDX almost completely prevented the thermal aggregation of MDH with no aggregation observed at one MDH/6 AtTDX molar ratio [16]. In the present study, the holdase chaperone activity of irradiated AtTDX was increased by at least two-fold compared to non-irradiated AtTDX (Figure 1B). Highest improvement in holdase chaperone activity was observed at a dose of 10 kGy (3–4-fold), but decreased further at higher doses (Figure 1B). In addition, we assayed foldase chaperone activity of AtTDX using the cysteine-free glucose-6-phosphate dehydrogenase (G6PDH) to differentiate foldase chaperone function of AtTDX from its thioredoxin function [18]. The spontaneous refolding (SR) of denatured G6PDH in the absence of AtTDX was ~16.1% of native G6PDH after 7 h renaturation (Figure 1C). In the presence of 1 μM non-irradiated (0 kGy) and irradiated (1, 5, 10, 20, 40 and 80 kGy) AtTDX proteins showed 41.2%, 40.8%, 41.6%, 37.4%, 41.7%, 43.7% and 41.7% refolding activity, respectively (Figure 1C). Thus, foldase chaperone activity of AtTDX was not affected by γ-irradiation.

### 2.2. Relationship between Structural Changes and Functions in γ-Irradiated AtTDX

**Figure 2:**
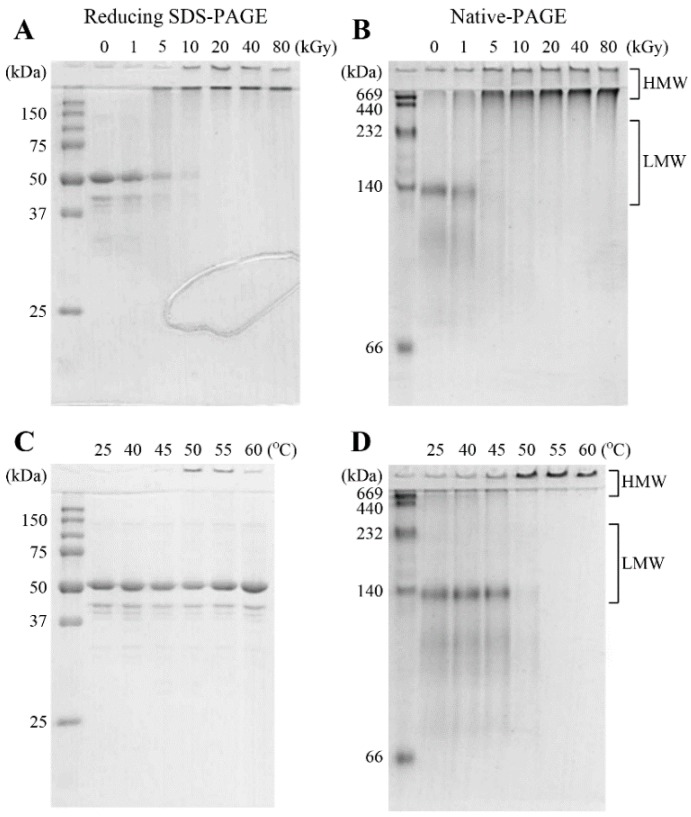
PAGE patterns of γ-irradiated or heat-treated AtTDX under denatured and native conditions. The γ-irradiated (**A**,**B**) or heat-treated (**C**,**D**) AtTDX proteins were separated by 12% SDS-PAGE under reducing condition (**A**,**C**) and 10% native-PAGE condition (**B**,**D**). The proteins were stained with Coomassie brilliant blue R-250. The numbers on the left side represent the molecular weights of the standard proteins.

The regulation of the multi-functionality of AtTDX is closely linked with its oligomeric status [16]. When exposed to γ-rays, the functional changes such as decreased thioredoxin activity with increased holdase chaperone activity might be due to the structural changes caused by γ-irradiation. To address/confirm the same, structural analyses of AtTDX were carried out using SDS- and native-polyacrylamide gel electrophoresis (PAGE) (Figure 2). The theoretical molecular weight of monomeric AtTDX is about 42 kDa. However, the monomeric size of the AtTDX protein was observed at about 52 kDa under reducing SDS-PAGE (Figure 2A,C). Like an Hsc70-interacting protein (Hip) described previously [19], AtTDX exhibited about 10 kDa greater molecular weight than predicted from its amino acid sequences. Several minor bands below 52 kDa were degraded forms of AtTDX. All PAGE analyses showed a similar pattern after γ-irradiation (Figure 2A,B). Under reducing SDS-PAGE condition, γ-irradiated monomeric AtTDX shifted towards γ-ray induced HMW complex structures (Figure 2A). Under native conditions without reducing agent and detergent such as dithiothreitol and sodium dodecyl sulfate, respectively, AtTDX protein showed various sized LMW forms (mainly tetrameric form) and HMW structures (Figure 2B).γ-Ray induced HMW complexes could not penetrate the separating PAGE gel, and also LMW forms and smeared LMW species were detected in SDS- and native-PAGE.

When the protein was exposed to heat for 30 min in a water incubator (Figure 2C,D), the protein shifted towards heat-induced HMW complexes above 50 °C under native conditions (Figure 2D), however, only the monomeric AtTDX form was detected up to 60 °C heat treatment in reducing SDS-PAGE (Figure 2C). This result suggests that HMW complexes can be generated by both γ-ray and heat treatment, but with distinguished organization patterns.

**Figure 3:**
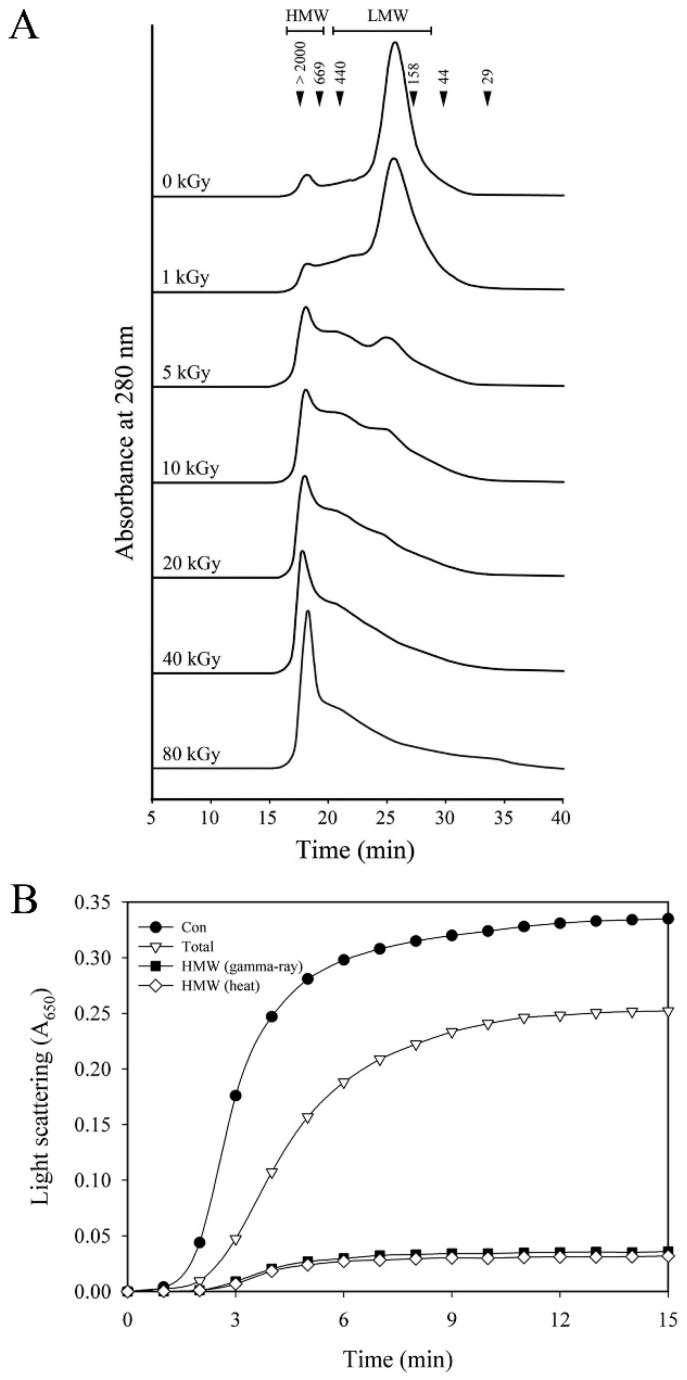
(**A**) SEC profiles of non-irradiated and irradiated AtTDX proteins. The numbers in the chromatogram represent the molecular weights of the standard proteins: blue dextran (>2000 kDa), thyroglobulin (669 kDa), ferritin (440 kDa), aldolase (158 kDa), ovalbumin (44 kDa), and carbonic anhydrase (29 kDa); (**B**) Holdase chaperone activities of γ-ray-induced or heat-induced HMW structure of AtTDX were measured using aggregation of MDH at 43 °C at a subunit molar ratio of 1 MDH/2 AtTDX. Total represents the total protein of AtTDX. HMW (γ-ray) represents the γ-ray-induced HMW structures of AtTDX protein. HMW (heat) represents the heat-induced HMW structures of AtTDX protein.

The structures of irradiated and non-irradiated AtTDX were also analyzed by size exclusion chromatography (SEC). Previously, we found that AtTDX could be separated into several distinct peaks corresponding to monomeric, dimeric, oligomeric, and HMW complexes [16]. LMW complex (including monomeric, dimeric, tetrameric, and hexameric) forms of AtTDX predominantly exhibited thioredoxin and foldase chaperone activities, whereas the holdase chaperone activity was found to be higher in double-layered dodecamer, and globular-shaped HMW complexes [16]. The SEC pattern of non-irradiated AtTDX showed a minor and major peak corresponding to HMW and LMW (mainly tetrameric form) complexes, respectively. Upon γ-irradiation, the concentration of γ-ray-induced HMW complexes of AtTDX increased with a concomitant decrease in LMW complex species (Figure 3A). These results were in good agreement with what we observed in native- and SDS-PAGE (Figure 2), confirming the γ-mediated structural changes in AtTDX depending of the doses of γ-irradiation.

To address the relationship between structural changes to HMW complexes by γ irradiation and AtTDX function as a holdase chaperone, we examined the holdase chaperone activity of γ-ray-induced HMW structures of AtTDX. The fraction corresponding to the γ-ray-induced or heat-induced HMW forms of AtTDX were separated by SEC. The γ-ray-induced HMW forms exhibited a higher holdase chaperone activity (about 3- to 4-fold) than that of the total protein, and similar results were seen with heat-induced HMW forms of AtTDX (Figure 3B). These results suggest that γ-ray-induced HMW of AtTDX have a strong holdase chaperone activity and is tightly associated with its holdase chaperone function.

### 2.3. Effect of γ-Irradiation on Hydrophobicity and Secondary Structure of AtTDX

**Figure 4:**
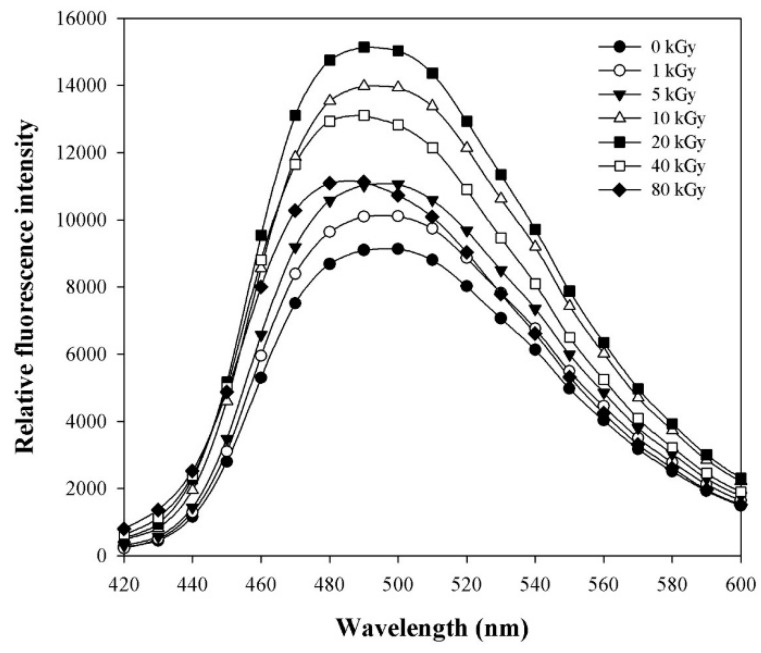
Change in hydrophobicity of AtTDX by γ-irradiation. Fluorescence spectra of bis-ANS bound to 100 μg/mL of each irradiated AtTDX protein. The control was measured in the absence of AtTDX protein.

Gamma-irradiation mediated changes in the hydrophobicity of AtTDX was observed by determining the binding of the 1,1′-bi(4-anilino)naphthalene-5,5′-disulfonic acid (bis-ANS) fluorophore, a widely used probe in the detection of hydrophobic regions on the surface of proteins [20]. The fluorescence levels of bis-ANS bound to irradiated AtTDX was significantly higher than that of non-irradiated AtTDX, reached its peak value at 20 kGy and decreased with any further increase in doses (Figure 4). In general, enhancement in the surface hydrophobicity is mainly associated with increased holdase chaperone activity of proteins [21]. In the present work, higher hydrophobicity of AtTDX at 10–20 kGy correlates with its better holdase chaperone activity observed at the same dose. Similarly, AtTDX irradiated with doses above 20 kGy minimized the exposure of hydrophobic domains and showed declined chaperone activity in comparison to 20 kGy-irradiated AtTDX, though they still showed improved holdase activity against non-irradiated AtTDX.

**Figure 5:**
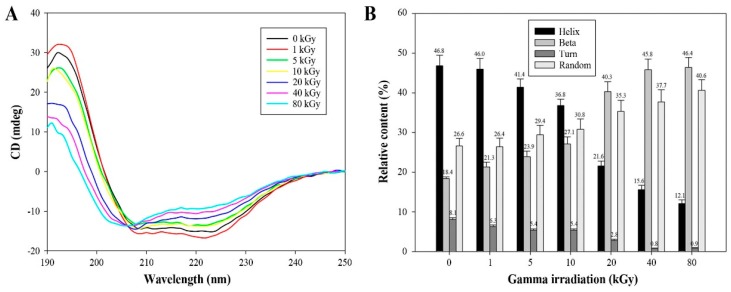
Change in the secondary structure of AtTDX by γ-irradiation. (**A**) Far-UV CD spectra of non-irradiated or irradiated AtTDX proteins in 10 mM Tris-HCl (pH 7.4) buffer. The ellipticity of the CD spectra was expressed in millidegrees (mdeg); (**B**) The comparison of the secondary structure index values (%) was based on the far UV-CD spectra results of AtTDX under different γ-irradiation doses. Data are the means of at least three independent experiments.

Far UV-circular dichroism (CD) spectra were used to ascertain the γ-irraditation mediated changes in the structure and function of AtTDX protein by estimating and comparing the secondary structural components between irradiated and non-irradiated protein (Figure 5). Figure 5A shows the original CD spectra of AtTDX protein exposed to different doses of γ rays. All the doses of γ rays have increased β- and random coil structures at the expense of α-helical and turn contents. Changes in secondary structural elements due to γ ray exposure were as follows: the β-sheet content increased from 18.4% to 46.4%; random coil content increased from 26.6% to 40.6%; α-helical content decreased from 46.8% to 12.1%; and the turn content decreased from 8.1% to 0.9%. These findings suggest that the γ-irradiated AtTDX increased the exposure of hydrophobic domains (Figure 4) with significant increase in exposure of β-sheet and random coil elements on the protein surface, while the exposure of α-helix and turn elements decreased (Figure 5).

## 3. Discussion

Multiple functions of AtTDX are known to be closely determined by its oligomeric status [16]. In present work, when AtTDX protein was exposed to γ rays, concentration of HMW complexes increased accompanied with decreased LMW protein species. In addition, smears of degraded protein molecules were also observed at higher doses. Similar dose-dependent effects of γ-irradiation (*i.e.*, protein oligomerization and fragmentation) have been reported in earlier studies [9,12,13,14,22,23,24]. Results of the present work suggests that the γ-ray-induced oligomerization of protein promotes the holdase chaperone activity of AtTDX, whereas, γ-ray-mediated dissociation or fragmentation triggers its thioredoxin and foldase chaperone functions. Protein exposed to γ-rays in the presence of water and oxygen, can lead to the formation of HMW complexes resulting from the generation of cross-links, electrostatic and hydrophobic interaction, and/or the formation of disulfide bonds [7,23,25]. In addition, the degradation of AtTDX might be caused by oxygen radicals generated by water radiolysis, resulting in both non-random and random fragmentation [26].

When the protein is exposed to high doses of γ-ray, the heating effect cannot be completely ruled out. In our γ-irradiation facility system, measuring a correct absorbed heat to samples was not possible due to inability to determine the exact heat absorption and loss during γ-irradiation. An absorbance dose of 10 kGy corresponds to a temperature rise of 2.4 °C in a food having the heat capacity of water [27]. When exposed to 80 kGy of γ rays at ambient temperature, the degree of exposure of protein is corresponding to a 44.2 °C. Therefore, the AtTDX protein was not subject to heat shock less than 80 kGy of γ irradiation because the conformation of AtTDX protein was not affected by heat stress less than 45 °C [16].

Heat- or γ-ray-induced HMW forms of AtTDX protein showed a strong holdase chaperone activity, but had a significant difference on SDS-PAGE analysis in reducing conditions. AtTDX monomer was detected with heat-induced HMW form of AtTDX by resulting reducing SDS-PAGE (Figure 2C), whereas γ-ray-induced HMW forms of AtTDX were gradually decreased in a monomer signal dose-dependently after γ-irradiation (Figure 2A). The SDS-PAGE data indicate that heat- and γ-ray-induced HMW complexes are totally distinct in their organization pattern. When exposed to heat shock, the HMW complex of AtTDX could be mainly formed by hydrophobic interaction. The γ-ray-induced HMW form of AtTDX might be formed by various factors such as chemical modification (oligomerization, cross-linking, carbonylation, disulfide bond, fragmentation or dissociation), electrostatic and hydrophobic interaction, and the secondary structural change of the proteins. Consequently, the structural changes of γ irradiated AtTDX could enhance holdase chaperone activity as well as maintain foldase chaperone activity and caused decreased thioredoxin activity. In light of available data, it is difficult to explain the exact biochemical mechanism for declined thioredoxin activity of AtTDX due to γ irradiation. The peroxidase (thioredoxin) activity of 2-Cys Prx and other similar proteins is known to be associated with their LMW form [13,28]. Therefore, γ-mediated structural transformation of AtTDX from LMW to HMW could be a possible reason for the gradual decline of thioredoxin activity of AtTDX in the present case. However, further experiments are warranted to precisely determine the biochemical/molecular basis for the γ-mediated changes in the thioredoxin activity of AtTDX protein. But based on the literature and also the present data, altered cysteine status seems to be implied more than thioredoxin fold changes for the declined thioredoxin activity in the present study. These results indicate that the physical properties of γ-irradiated AtTDX might be changed by γ irradiation but not by heat stress.

## 4. Experimental Section

### 4.1. Materials

Insulin, DTT, ampicillin (Amp), imidazole, l-rhamnose, hydrogen peroxide (H_2_O_2_; 30% *v*/*v*), nicotinamide adenine dinucleotide phosphate reduced (NADPH), porcine heart mitochondrial malate dehydrogenase (MDH), the Cys-free form of G6PDH, glucose-6-phosphate (G6P), nicotinamide adenine dinucleotide phosphate (NADP), bovine serum albumin (BSA), and SDS were purchased from Sigma (St. Louis, MO, USA). Protein molecular size standards used in PAGE were obtained from ELPIS (Daejeon, Korea). Bis-ANS was obtained from Molecular Probes (Invitrogen Corporation, Carlsbad, CA, USA).

### 4.2. Expression and Purification of Recombinant AtTDX Protein

To express the recombinant AtTDX protein, the *AtTDX* gene was cloned into a pET28a vector (Novagen, Gibbstown, NJ, USA) and the resultant plasmid was transformed into *E. coli* strain, BL21 (DE3). Talon metal affinity resin (Clontech Laboratories Inc., Mountain View, CA, USA) used to purify the His-tagged protein following the method as described previously with slight modifications [29] and eluted by thrombin cutting. Purified AtTDX protein dialyzed into 50 mM Tris-HCl (pH 8.0) was used for biochemical analysis. The concentration of protein was determined according to Bradford method using BSA as a standard [30].

### 4.3. γ-Ray Irradiation

AtTDX protein (2 mg/mL) was divided into 1 mL aliquots in sterilized of snaplock microtube of 1.7 mL (Axygen, Union City, CA, USA), and the samples were exposed to γ rays at ambient temperature using a high-level cobalt-60 irradiator (point source AECL, IR-79, MDS Nordion International Co., Ltd., Ottawa, ON, Canada) at the Advanced Radiation Technology Institute (ARTI), Korea Atomic Energy Research Institute (KAERI) with absorbed doses ranging from 1 to 80 kGy as previously described [31].

### 4.4. PAGE and SEC

Native-PAGE and reducing SDS-PAGE were performed as previously described [28]. SEC analyses of AtTDX proteins were performed using fast protein liquid chromatography (FPLC) (AKTA; Amersham Biosciences, Uppsala, Sweden) at 4 °C with a Superdex 200 10/300 GL column. The column was equilibrated with 50 mM HEPES buffer (pH 8.0) having 100 mM NaCl to a flow rate of 0.5 mL/min at 4 °C.

### 4.5. Thioredoxin Activity Assay

Thioredoxin catalysis of insulin reduction was spectrophotometrically measured at 340 nm by observing turbidity due to precipitation of the free insulin β-chain, as described previously with slight modifications [32]. The assay mixture comprised of 100 mM KH_2_PO_4_, 2 mM EDTA, 0.1 mg insulin, 1 mM DTT, and 10 μM of γ-irradiated or non-irradiated AtTDX proteins. Activity was monitored for 30 min at 25 °C on a spectrophotometer (Evolution 300 UV-VIS spectrophotometer; Thermoscientific, Worcester, MA, USA).

### 4.6. Molecular Chaperone Activity Assay

Holdase chaperone activity was measured by determining the prevention of the thermal aggregation of heat-sensitive MDH (substrate) by AtTDX. MDH was incubated in a 50 mM HEPES-KOH (pH 8.0) buffer at 43 °C with varying levels of AtTDX or 2 μM of γ-irradiated AtTDX. Thermal aggregation of the substrate was recorded at 650 nm for 15 min on a spectrophotometer. Foldase chaperone activity was analyzed using 4 M guanidine hydrochloride (Gdn-HCl)-denatured G6PDH (substrate) with slight modifications in the procedure procedure elaborated previously [16,18]. Denatured G6PDH (2 μM) was diluted 500-fold in a 50 mM Tris-HCl buffer (pH 7.5), and refolding activity was determined in terms of G6PDH activity.

### 4.7. Measurement of Hydrophobicity

Protein hydrophobicity was monitored by determining bis-ANS fluorescence with an Infinite M200 (Tecan Group Ltd., Männedorf, Switzerland). After addition of 10 μM bis-ANS to protein samples (100 μg) pre-treated at room temperature for 30 min, the binding of bis-ANS was measured. Bis-ANS fluorescence excitation was set at 380 nm, and the emission was recorded between 400 and 600 nm [20].

### 4.8. CD Spectroscopy

Pre- and post-γ irradiated AtTDX in 10 mM Tris-HCl (pH 7.4) buffer was used for UV-CD spectral analysis with a Jasco J-815 spectropolarimeter (Jasco Co., Great Dunmow, UK). All measurements were made between 190 and 250 nm with 1 nm bandwidth, 1 s response time, and 100 nm/min scanning speed. The spectra were collected and corrected by subtracting a blank containing 10 mM Tris-HCl (pH 7.4) buffer, reduction of noise, and smoothing. The program JASCO Canvas was used for the measurement of the percentage of secondary structure of non-irradiated and irradiated AtTDX proteins following the method given by Yang *et al.* [33].

## 5. Conclusions

Holdase chaperone activity of AtTDX can be improved using γ-irradiation up to a dose of 10 kGy, whereas foldase chaperone activity was not affected by γ-irradiation. The functions of AtTDX were closely linked with its structural conditions, including oligomerization, fragmentation or dissociation, increasing hydrophobicity, and the secondary structural changes of the protein. The modification methods for improving holdase chaperone activity without changing foldase chaperone activity of such proteins involve high cost, time, and complicated efforts. However, radiation technology could be considered as a fast, easy, and cost-effective tool to enhance or maintain chaperone activity of thioredoxin-like protein.
